# The Voice of Emotion across Species: How Do Human Listeners Recognize Animals' Affective States?

**DOI:** 10.1371/journal.pone.0091192

**Published:** 2014-03-12

**Authors:** Marina Scheumann, Anna S. Hasting, Sonja A. Kotz, Elke Zimmermann

**Affiliations:** 1 Institute of Zoology, University of Veterinary Medicine Hannover, Hannover, Germany; 2 Department of Neuropsychology, Max Planck Institute for Human Cognitive and Brain Sciences, Leipzig, Germany; 3 Day Clinic for Cognitive Neurology, University Hospital Leipzig, Germany; 4 School of Psychological Sciences, The University of Manchester, Manchester, United Kingdom; Utrecht University, Netherlands

## Abstract

Voice-induced cross-taxa emotional recognition is the ability to understand the emotional state of another species based on its voice. In the past, induced affective states, experience-dependent higher cognitive processes or cross-taxa universal acoustic coding and processing mechanisms have been discussed to underlie this ability in humans. The present study sets out to distinguish the influence of familiarity and phylogeny on voice-induced cross-taxa emotional perception in humans. For the first time, two perspectives are taken into account: the self- (i.e. emotional valence induced in the listener) versus the others-perspective (i.e. correct recognition of the emotional valence of the recording context). Twenty-eight male participants listened to 192 vocalizations of four different species (human infant, dog, chimpanzee and tree shrew). Stimuli were recorded either in an agonistic (negative emotional valence) or affiliative (positive emotional valence) context. Participants rated the emotional valence of the stimuli adopting self- and others-perspective by using a 5-point version of the Self-Assessment Manikin (SAM). Familiarity was assessed based on subjective rating, objective labelling of the respective stimuli and interaction time with the respective species. Participants reliably recognized the emotional valence of human voices, whereas the results for animal voices were mixed. The correct classification of animal voices depended on the listener's familiarity with the species and the call type/recording context, whereas there was less influence of induced emotional states and phylogeny. Our results provide first evidence that explicit voice-induced cross-taxa emotional recognition in humans is shaped more by experience-dependent cognitive mechanisms than by induced affective states or cross-taxa universal acoustic coding and processing mechanisms.

## Introduction

The recognition of affective information in human voice plays an important role in human social interaction and is linked to human empathy, which refers to the capacity to perceive, understand and respond to the unique affective state of another person (e.g., [1], [2]). Human speech and human non-linguistic vocalizations convey emotional states in the form of prosodic cues (e.g.[3], [4], [5], [6]). Based on these prosodic cues humans are able to recognize the emotional state of other humans (e.g., [7], [8], [9], [10]). This is termed voice-induced emotional recognition. Cross-cultural studies demonstrated that humans with different linguistic backgrounds exhibit many similarities in terms of how they express and identify emotions in human voices and music (e.g., [11], [12], [13], [14], [15]). This may suggest that affective prosodic components in humans are predominantly organized by innate mechanisms and may have derived from a pre-human origin [6].

More than 130 years ago Darwin [16] postulated in his masterpiece “The Expression of Emotion in Man and Animals” that emotional expressions evolved continuously from animals to humans. Current empirical research revealed cross-taxa similarities in the acoustical conveyance of emotions across different mammalian groups (e.g., [17], [18], [19], [20], [21], [22], [23]) which show similarities to prosodic cues in human speech and non-verbal vocalizations (e.g., [3], [6], [24], [25]). Various models were developed to characterize the universal relationship between the structure of vocalizations and their emotional content (e.g., [26], [27], [28], [29]). Playback studies on cross-taxa recognition demonstrated that humans were able to classify heterospecific vocalizations to the respective recording contexts (e.g. [30], [31], [32], [33]) and that non-human animals showed adequate behavioral responses to vocalizations of heterospecific senders, suggesting voice-induced cross-taxa recognition (e.g., [34], [35], [36]).

The question whether the perception of the sender's emotions is based on the self-induced emotions in the listener (e.g., I feel afraid when hearing this sound, therefore I think the sender is afraid, too) or on learned associations between the sound and the context (e.g. I know this is a sound emitted in an agonistic context, therefore I think the sender is afraid), motivates a distinguished view on two perspectives: the self-perspective, i.e. which emotion the vocalization induced in the recipient, *versus* the others-perspective, i.e. whether recipients are able to classify the affective information of the respective vocalization correctly. Previous studies on voice-induced cross-taxa emotional recognition focused solely on the others-perspective. It was shown that human listeners are able to recognize the context and/or its emotional content in which the animal was calling (cats: [37]; dogs: [30], [31], [38], [39], [40], [41], [42], [43]; pigs: [32]; macaques: [33]; except [7]). Since most of the studies confronted human listeners with only one species, either domesticated (to some extent familiar to human listeners) or primate (phylogentically close related to human listeners), it still remains unclear whether voice-induced cross-taxa emotional recognition can be explained by familiarity or by phylogeny as a result of cross-taxa universal acoustic coding and processing mechanisms. In a recent functional magnetic resonance imaging study Belin et al. [7] tested Darwin's continuity hypothesis, investigating human brain activations in response to human, non-human primate (rhesus monkey) and non-primate (cat) vocalizations recorded in affective contexts of positive or negative emotional valence. Comparable human brain areas were activated when listening to affective human and animal vocalizations, speaking in favor of phylogenetic universals [7]. However, humans were not able to recognize the emotional valence of animal vocalizations. Lack of recognition of the emotional valence of cat vocalizations was surprising in light of previous findings by Nicastro and Owren [37]. The authors argued that the discrepancy may be explained either by cognitive components (i.e. human listeners recognized human laughter and attributed it to the positive valence category), or by the fact that subjects' familiarity with cats had not been controlled. Thus, it remains open to what extent cross-taxa recognition of the emotional state of the sender is triggered by acoustic stimuli familiarity or by phylogenetic relatedness to the tested species.

The present paper contrasts for the first time the effect of familiarity and phylogeny on voice-induced cross-taxa emotional recognition, while simultaneously taking into account both the self- and the others-perspective. We used agonistic and affiliative voices of human infants (conspecific control) and three animal species varying in their degree of familiarity and phylogeny to humans: (1) dogs – very familiar but phylogentically distant to humans, (2) chimpanzees – less familiar but phylogenetically close to humans and (3) tree shrews – unfamiliar and phylogenetically distant to humans. To prove the above mentioned assumption concerning familiarity we used an objective measurement ( =  objective familiarity) where the participants had to label the sound spontaneously (at this stage of the rating they did not even know they were only listening to living beings). We will report to which extent voice-induced cross-taxa emotional recognition exists (others-perspective) and can be explained by the following three factors: (1) the self-perspective as a reflection of the induced affective state, (2) the familiarity with the acoustic stimuli and (3) the phylogenetic distance to the animal species. If only a reflection of the self-perspective is required for voice-induced cross-taxa emotional recognition (e.g., I am afraid therefore I think the animal is afraid too), we expected no difference between the perspective on the valence ratings. If familiarity is required for voice-induced cross-taxa vocal recognition, we expected a high recognition accuracy of emotional valence for all playback categories which were assigned to the correct species, whereas we expect a low recognition accuracy for all playback categories where human listeners did not label the correct species. If phylogeny plays a role in voice-induced cross-taxa vocal recognition, we expected a high recognition accuracy of emotional valence for humans and chimpanzees but not for dogs and tree shrew voices.

## Materials and Methods

### Ethical Statement

The experiment was conducted with the approval of the ethics committee of the University of Leipzig and in compliance with the Declaration of Helsinki. The participants gave written consent and received 7 Euros per hour as compensation for their efforts.

### Participants

Twenty-eight healthy childless male participants aged 21–28 years (mean = 24.21±2.01 years) took part in the study. It is known from the literature that men and women show different brain responses to human infant vocalizations [9] and adult emotional speech [44], [45], [46]. To ensure that this confounding factor did not affect our results, the present study focused on male participants only. The participants did not own a dog at the time of testing.

### Acoustic stimuli

**Figure 1:**
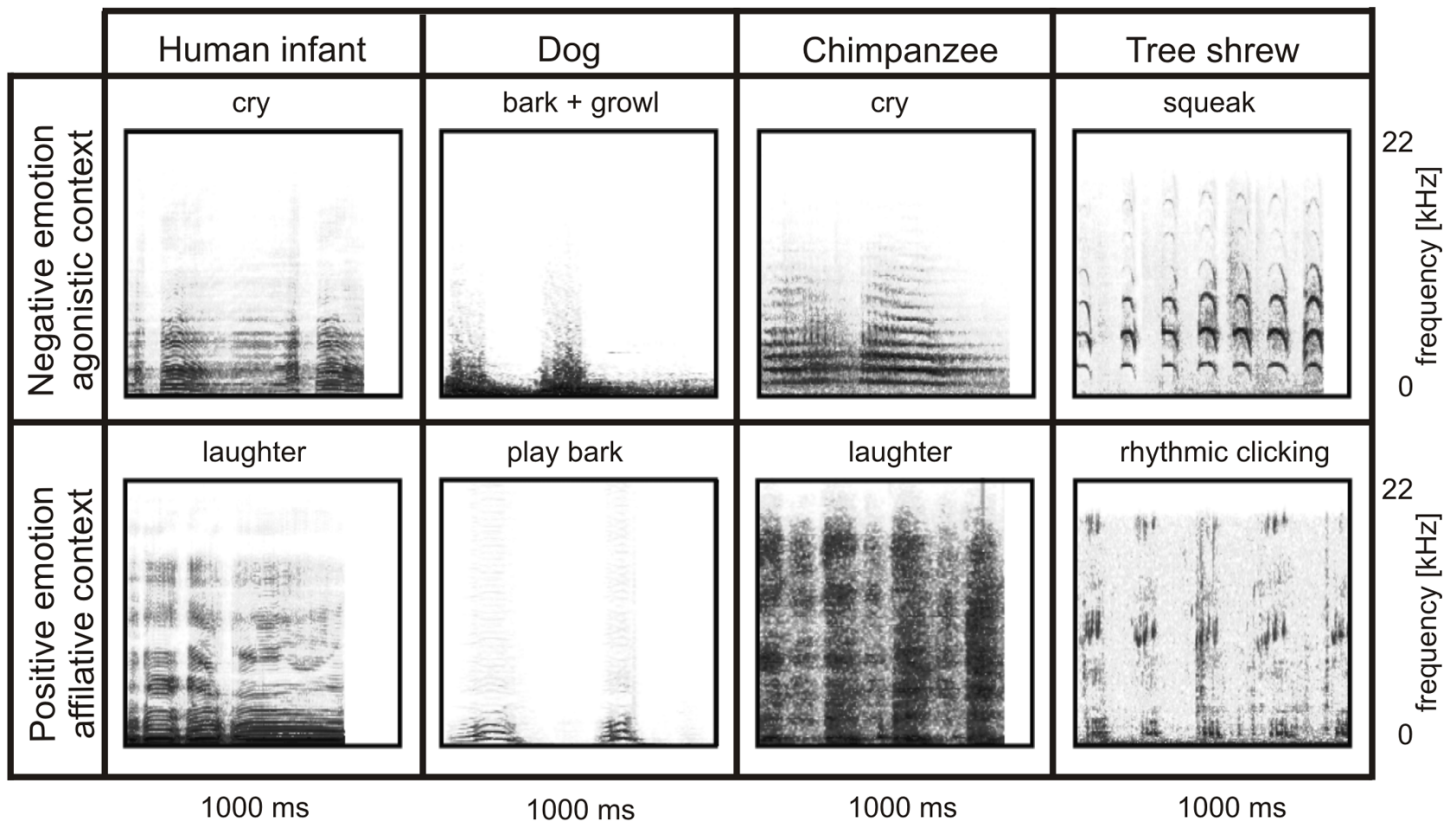
Sonograms of examples of playback stimuli of the eight playback categories.

We used 192 recorded acoustic stimuli of four species (human infant, dog, chimpanzee and tree shrew) in two distinct superordinate context categories (agonistic versus affiliative; for detailed context description see Table 1, Figure 1) as playback stimuli. Because we recorded animals in natural contexts, it was essential to use also for human stimuli spontaneously produced vocalizations recorded in natural contexts (also because studies already showed differences in the vocal production as well as in the perception of play-acted and authentic vocalizations [47], [48]). We chose vocalizations of human infants as human stimuli because adults will be aware that they are being recorded. Thus cognitive processes can alter spontaneously produced vocalizations.

**Table 1:** Description of playback categories.

Playback category	no. of senders	Recording conditions	detailed context description (Calls were recorded during…)
**Agonistic context category – negative emotional valence**			
Human infant	5	MS at PH: SK6 + MPR	situations where the mother forbids the infant something (e.g., toy, food, access).
dog	7	MS at PH: SK6 + MPR	aggressive conflicts between dogs (another dog approached the sender, the sender vocalized and the other dog went away or changed its behaviour; sender defends a toy) or in a stranger situation (a strange person approached a leashed dog which barked and showed its teeth). Eight of the calls occurred during human-dog and 16 of the calls during dog-dog interactions.
chimpanzee	8	MS at ZS: SK6 + MPR	aggressive interactions between chimpanzees. Chimpanzees chased each other in the enclosure and had physical conflicts.
tree shrew	6	SS and WK at IZ: SME64 + PA + LP	the sender produced the calls while chasing the other away [57].
**Affiliative context category – positive emotional valence**			
Human infant	5	MS at PH: SK6 + MPR	tickling or playing from 1 ½ year-old infants.
dog	8	MS at PH: SK6 + MPR	play interactions: either dogs played with each other (chasing each other but also waiting that the other follows; barking in front of another dog to initiate play behavior), or a human held a toy (e.g., ball or stick) in front of the dog. Eight of the calls occurred during human-dog and 16 of the calls during dog-dog interactions.
chimpanzee	6	MS at ZS: SK6 + MPR; BF at ZH and SP: SMKH 816 + Nagra IV-SJ	tickling sessions of five chimpanzee infants and one adult chimpanzee.
tree shrew	6	SS and WK at IZ: SME64 + PA + LP	male-female interaction. The male is producing these calls to attrack an oestric female [58].

Information on number of senders, recording conditions (recorder, place of recording and equipment) and detailed context description for each playback category. All stimuli (except affiliative chimpanzee stimuli obtained by Birgit Fördereuther which were recorded during tickling sessions) were videotaped. For the tree shrew calls, which were obtained by Simone Schehka and colleagues, we refer to their video analyses for context classification [57], [58]. For our own recordings we synchronized audio and video recordings and assigned each vocalization to a detailed context. Each detailed context was assigned to one of the two superordinate context categories, affiliative or agonistic context.

Abbreviations: recorder: MS  =  M. Scheumann, SS  =  S. Schehka, WK  =  W. Konerding, BF  =  B. Förderreuther; place of recording: PH  =  private households, ZS  =  Leintal Zoo Schwaigern, IZ  =  Institute of Zoology, University of Veterinary Medicine Hannover, ZH  =  Hannover Zoo, SP  =  Schwaben Park; recording equipment: SK6  =  Sennheiser microphone K 6, MPR  =  Marantz pocket recorder PMD 660, SME64  =  Sennheiser microphone ME64, PA  =  pre-amplifier (Avid Technology, Öhringen, Germany, M-Audio DMP3), LP  =  laptop (Toshiba, Irvine, CA, USA, Satellite A10-s100) equipped with a A/D converter: (National Instruments, Austin, TX, USA, DAQCard-6062E) and the software NIDisk (Engineering Design, Belmont, MA, USA), SMKH 816  =  Sennheiser microphone MKH 816.

For each species and each context category 24 playback stimuli were used containing single calls or call sequences from 5 to 8 different senders. A call was defined as one continuous sound element. The agonistic context category was classified as an emotionally negative context ( = negative emotional valence). In this context category calls were produced in conflict situations to change or finish a current interaction (e.g., distance between interaction partners increased). The affiliative context category was classified as an emotionally positive context ( = positive emotional valence). In this context category calls were produced to maintain the current situation (e.g., small distance between interaction partners). All in all, we tested stimuli of four species linked to two emotional valences (8 playback categories) termed: agonistic human infant, affiliative human infant, agonistic dog, affiliative dog, agonistic chimpanzee, affiliative chimpanzee, agonistic tree shrew and affiliative tree shrew.

In preparing the playback stimuli, we selected calls of a single sender of good signal-to-noise ratio and standardized the duration to approximately 1 second (Table 2) by selecting natural calls or call sequences matching the standardized duration as well as possible using the software SIGNAL 3.1 (Engineering Design, Berkeley, California, U.S.A.). All playback stimuli were sampled with a sample frequency of 44.1 kHz (16 bit, mono). Sound intensity was normalized to 60 dB using PRAAT (www.praat.org; [49]).

**Table 2:** Acoustic characterization of the playback categories.

	Human infant	Dog	Chimpanzee	Tree shrew
**Agonistic context – negative emotional valence**				
STIM DUR	0.72±0.18	0.85±0.15	0.75±0.18	0.77±0.13
CALL DUR	87.54±18.82	72.01±20.10	100±0	59.98±5.15
No. CALL	1.79±1.32	2.58±1.18	1±0	6.38±1.21
PEAK	1241.89±738.20	773.23±282.51	1815.80±444.92	5279.18±1164.82
MEAN f0	510.73±145.93	358.35±224.62	1197.87±158.67	2341.90±282.48
SD f0	81.11±86.05	69.48±68.51	73.76±49.76	194.36±100.96
%VOI	76.97±25.67	42.32±32.38	92.83±9.19	45.35±11.47
**Affiliative context – positive emotional valence**				
STIM DUR	0.76±0.13	0.72±0.16	0.82±0.14	0.69±0.16
CALL DUR	88.64±14.28	55.34±12.66	64.47±10.14	54.94±14.51
No. CALL	2.21±1.41	2.29±0.69	4.38±1.81	5.04±2.10
PEAK	896.97±731.50	312.65±426.50	799.56±359.07	438.09±1287.29
MEAN f0	479.66±323.51	493.47±216.22	-	-
SD f0	110.28±177.85	68.89±49.61	-	-
%VOI	78.86±19.12	39.86±24.35	0±0	0±0

Mean ± standard deviation of following acoustic parameters: STIM DUR  =  duration of the stimulus (seconds), CALL DUR  =  percentage of time of the calls in the whole stimulus (%), No. VOC  =  number of calls in the stimulus; PEAK  =  peak frequency (Hz), MEAN f0  =  mean fundamental frequency (f0, Hz), SD f0  =  standard deviation of f0 (Hz), %VOI  =  percentage of voiced frames in the stimulus (%).

For the acoustic characterization of the playback stimuli an acoustic analysis using PRAAT and SIGNAL 3.1 was performed. For each playback stimulus, the following measurements were obtained: duration of the stimulus sequence (STIM DUR), percentage of time of the call in the stimulus sequence (CALL DUR), number of calls in a stimulus sequence (No. CALL), peak frequency (PEAK), mean fundamental frequency (MEAN f0), standard deviation of the fundamental frequency (SD f0) and percentage of voiced frames (%VOI). The mean values for each playback category and each measurement are listed in Table 1.

### Experimental Set-up

Each participant was tested separately in a quiet, dimmed room. Stimuli were presented via headphones (Audio-Technica ATH-M40fs). The instructions and the computerized rating were presented via a PC with a 17 inch monitor. The participant responded to the questions by pressing a button on a five button box (EX-Key Keyboard Logic) or by speaking into a microphone. Visual and acoustic responses were recorded by a video camera (CCD Camera AV Tech; Panasonic DVD Recorder DMR-EH52). The experimenter sat in the same room behind a visual barrier, observing the participant via a monitor and typing the spoken responses into a laptop.

### Experimental Task

Each participant listened to all 192 playback stimuli in a randomized order twice in two blocks. Each block was divided into 4 segments of 48 stimuli. Between the segments the participant was free to take a break. The sound level was the same for each participant. The Self-Assessment Manikin Scale (SAM; 5-point scale), a standardized scale for emotional ratings, was used as an intuitive classification task [50]. In the first block participants were instructed to indicate what they felt when listening to the sound without any prior information about the nature of the sound ( = self-perspective). We analyzed the following three questions in the first block: (1) Participants had to rate the valence of the sound on a 5-point scale (valence SAM scale; [50]) ranging from very negative to very positive ( = self-valence, “How does the sound make you feel?”) by pressing a button on the five-button box. The direction of the scale was alternated and counterbalanced between participants. (2) Participants had to rate how familiar the sound was on a 5-point scale ranging from unfamiliar to very familiar ( = assumed familiarity, “Is the sound 1 (familiar), 2, 3, 4, 5 (unfamiliar) to you?), again by pressing a button on the five-button box. (3) Participants were asked to label the sound by speaking into the microphone ( = objective familiarity, “What kind of a sound was it?”). Note that at this stage of the rating participants did not even know they were only listening to human and animal voices.

The second block aimed at obtaining the others-perspective. Participants listened to the same 192 acoustic stimuli in the same order as in the first block, but this time participants were informed that all sounds were voices of living beings and were instructed to rate what the animal was feeling while calling. We analyzed the participants' valence rating where participants had to rate the emotional valence of the call on a 5-point scale (valence SAM scale) ranging from very negative to very positive ( = others-valence, “What is the situation like in which the animal is calling?”) by pressing a button on the five-button box. The direction of the scale was again alternated and counterbalanced between participants.

After finishing the playback experiment, participants filled out a paper-and-pencil questionnaire including the question how much time participants spent with human infants, chimpanzees, dogs or tree shrews on a 5-point scale ranging from “never” to “very often” ( = interaction time).

### Data preparation

To test our assumption about familiarity of human listeners to the vocalizations of the used species and call type/recording context, we obtained the objective familiarity by calculating the species recognition index as the percentage of responses where the calling species was correctly recognized: number of correctly labelled stimuli divided by the total number of stimuli for each playback category and participant. The following labels were defined as correct responses: (1) for human infants – infant/baby, child, human, man or woman (2) for dogs – dog, bark (3) for chimpanzees – monkey/ape, primate, chimpanzee (4) for tree shrew – tree shrew, tupaia, Scandentia (see also Table S1). We classified playback categories as familiar if more than 70% of the stimuli where assigned to the correct species.

To assess the assumed familiarity we calculated the mean scores (1–5;  =  assumed familiarity index) of the assumed familiarity rating for each participant and each playback category. To analyze whether a playback stimulus induced emotional responses in human listeners ( =  self-valence) and how human listeners classified the emotional valence of the context of the sender ( =  others-valence), we transformed the 5-point SAM scale into the following scores: -2 (very negative), -1 (negative), 0 (neutral), 1 (positive) and 2 (very positive). The mean score for the valence ratings (−2 to 2;  =  valence index  =  VI) was calculated for each participant, each playback category and each perspective separately.

### Statistical analysis

To make sure that there was no boredom/exhaustion effect while listening to 192 stimuli, i.e. that at the end of a block the participants became tired and just pressed the button for neutral response and did not put effort in real assessment of the emotional valence of the voices, we calculated the percentage of stimuli for which participants pressed the neutral button across the four segments of each block. For both the first and the second block the percentage across the sessions within a block was quite similar (block 1: 36.93–43.89%); block 2: 29.28%–32.14%). Further we compared the valence indices for each playback category between the four segments of each block. Since there were no significant differences between the four segments for all playback categories for the VI_self_ (F≤2.44, df = 3, N = 28, p≥0.070) and after applying a Bonferroni correction also not for VI_other_ (F≤1.72, df = 3, N = 28, p≥0.170 for all playback categories except for HN: F = 3.44, df = 3, N = 28, p = 0.021; p_corr_  =  0.168), we used all acoustic stimuli for further analysis.

There are various approaches to assess emotional reactions to stimuli using discrete emotional categories (e.g., fear, anger, happiness etc.) or dimensional states [51]. In this study we choose the valence rating as an intuitive classification task to limit cognitive associations with the acoustic stimuli and the species or context. The valence rating was then used as a measurement for classification. Thus, we assumed that negative valence scores indicate a classification as an induced negative emotion (self-perspective) or as a negative emotional context ( = agonistic context; others-perspective) whereas positive valence scores indicate a classification as an induced positive emotion (self-perspective) or an classification as an positive emotional context ( = affiliative context; others-perspective). For the others-perspective we defined that participants recognized the emotional valence of a playback category correctly if the rated valence matched the assumed emotional valence of the recording context.

The fact that our assumption concerning species familiarity was not entirely supported by the objective familiarity measurement precluded the use of familiarity and phylogeny as two orthogonal factors in a repeated measurement ANOVA design. To account for this, we used the more general factor, species. Thus, we calculated a two-factorial repeated measurement ANOVA using the factors context (levels: agonistic and affiliative) and species (levels: human infant, dog, chimpanzee and tree shrew) to analyze the effects of context and species on valence ratings ( = VI). If the Mauchly's test indicated that the assumptions of sphericity are violated (p≤0.05), we corrected the degrees of freedom using Greenhouse-Geisser estimates of sphericity [52]. Using a one-sample t-test we tested whether the valence index was significantly different from zero. We defined a playback category as: (1) emotionally positive if the valence index was positive and significantly differed from zero, (2) emotionally negative if the valence index was negative and significantly differed from zero and (3) neutral if the valence index did not significantly differ from zero. Because of multiple testing we corrected the p-values of the one-sample t-test using a Bonferroni correction (p_corr_). To investigate the effect of perspective on valence indices we conducted a three-factorial repeated measurement ANOVA using the factors: perspective (levels: self-perspective and others-perspective), context (levels: agonistic and affiliative) and species (levels: human infant, dog, chimpanzee and tree shrew). Further we conducted dependent t-tests comparing the self- with the others-perspective for each playback category and corrected the p-values using a Bonferroni correction (p_corr_).

To investigate the influence of familiarity and of self-perspective on cross-taxa emotional recognition we calculated the emotional correct assignment index (ECI) based on the valence rating of the others-perspective for each of the eight playback categories using the following formula: (1) for the playback categories of the negative context  =  (number of playback stimuli with negative scores)/(total number of playback stimuli); (2) for playback categories of the positive context =  (number of playback stimuli with positive scores)/(total number of playback stimuli). The ECI was correlated with the means for the species recognition index (objective familiarity), the interaction time, the assumed familiarity and the VI_self_ using a Pearson correlation across the eight playback categories.

All tests were performed using the statistical software SPSS 21. Bonferroni correction was calculated using an SPSS syntax according to the formula p_corr_  =  p-value * number of tests.

## Results

### Testing the assumption of species familiarity towards human listeners

**Figure 2:**
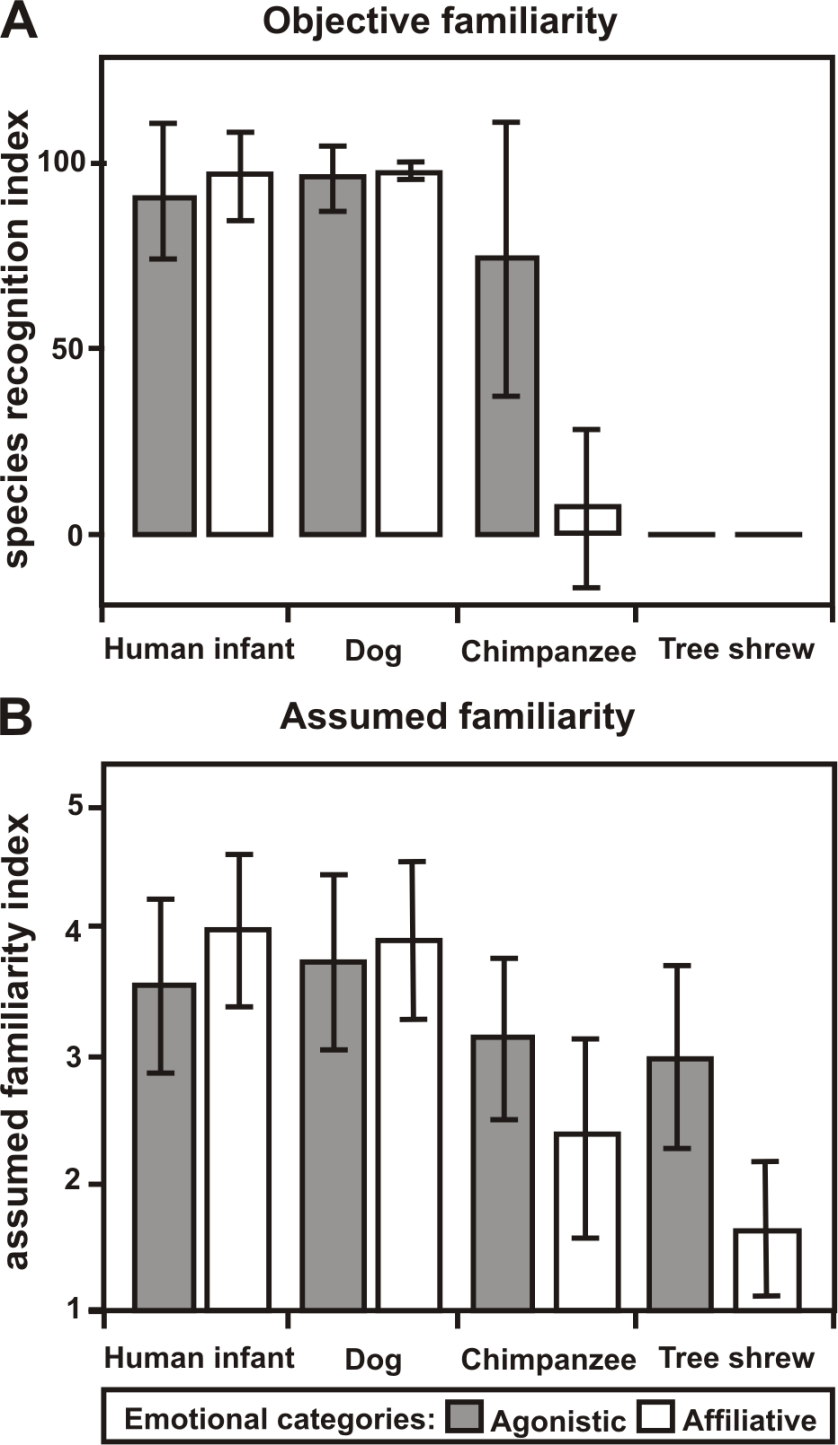
Familiarity ratings for the playback categories. Mean and standard deviation of the (A) species recognition index and the (B) assumed familiarity index for each playback category.

Based on the objective familiarity rating a two-factorial repeated measurement ANOVA revealing significant main effects of context (F = 55.89, df = 1, N = 28, p<0.001) and species (F = 383.96, df = 1.66, N = 28, p<0.001) but also a significant interaction between both (F = 98.51, df = 1.1, N = 28, p<0.001; Figure 2a). This indicates that the context had different effects on the objective familiarity rating depending on the species. Human infant and dog stimuli showed the highest percentage of correct recognition (≥91.93%; Figure 2a), whereas no participant recognized the tree shrew stimuli correctly. Agonistic tree shrew stimuli were mostly associated with birds (53.57% as a result of bird/chirp: 44.64%, N = 20, sea-gull: 4.61%, N = 2, parrot: 3.57%, N = 1 and blackbird/chick: 0.75%, N = 1) or Rodentia (25.15%; as a result of mice: 18.6%, N = 8, rodent: 2.38% N = 3, guinea pig: 2.83% N = 1 and others 1.34% N = 3), whereas affiliative tree shrew stimuli were either associated with inanimate objects (38.99% as a result of sounds of a horse-drawn carriage: 11.76% N = 12, sounds of vehicles: 7%. N = 6, noise from the street: 7% N = 5, sounds of a machine: 2.08%, N = 7 and others: 11.16%, N = 28) or participants reported that they had no idea of the nature of the sound (46.73%; for more information see also Table S1). Interestingly, while participants labelled 75.89% of the agonistic chimpanzee voices as a primate, they only labelled 7.44% of the affiliative chimpanzee voices as a primate. Notably, the percentage of correctly labelled affiliative chimpanzee voices mostly relies on one participant (without this participant the percentage even dropped to 4.01%). This participant was also the only one who specified the species chimpanzee for 3 stimuli of the affiliative chimpanzee playback category.

Based on these results, we had to correct our assumptions of familiarity. Thus, we classified agonistic and affiliative human infant and dog voices as well as agonistic chimpanzee voices as familiar and affiliative chimpanzee voices and agonistic and affiliative tree shrew voices as unfamiliar.

Statistical analysis of the assumed familiarity rating also revealed significant main effects of context (F = 20.26, df = 1, N = 28, p<0.001) and species (F = 100.12, df = 2.1, N = 28, p<0.001) but again also a significant interaction between both (F = 53.788, df = 2.2, N = 28, p<0.001; Figure 2b). Comparing the results for the objective and assumed familiarity rating we found slight discrepancies. For example, although both tree shrew voices could not be correctly labelled, participants rated agonistic tree shrew voices as more familiar than affiliative tree shrew voices. Agonistic tree shrew voices received a middle sized assumed familiarity score of 2.98, indicating that participants assumed to be familiar with animal voices but were not able to recognize them correctly.

### Self-perspective

**Figure 3:**
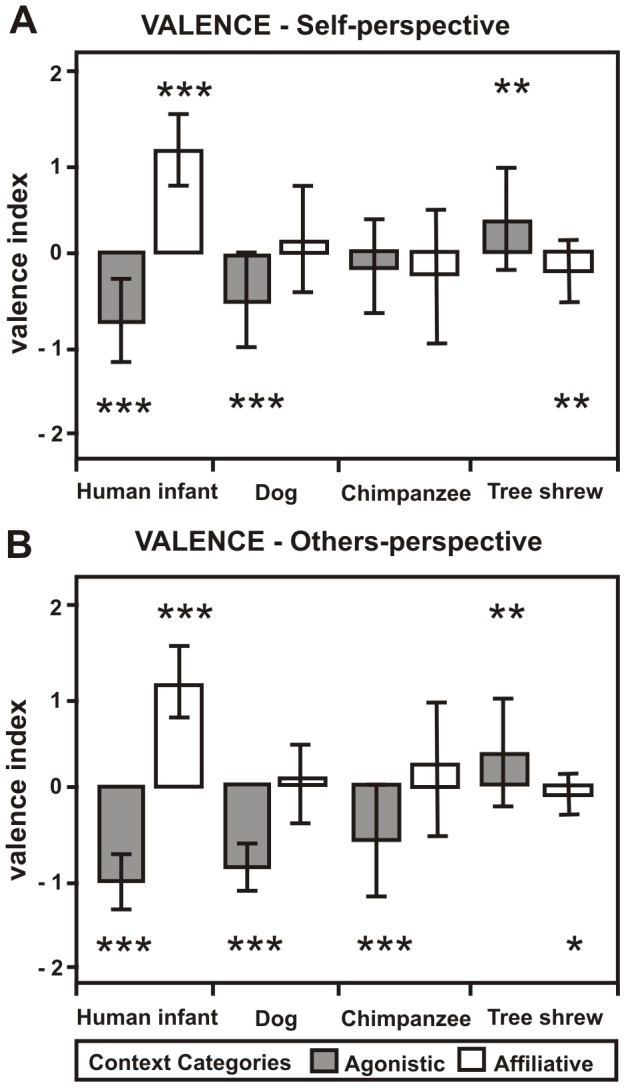
Valence ratings for the playback categories. Mean and standard deviation of the valence index for the playback categories of the (A) self-perspective and the (B) others- perspective. one-sample t-test *** p≤0.001, **p≤0.01, *p≤0.05.

The two-factorial repeated measurement ANOVA revealed significant main effects of context (VI_self_: F = 48.01, df = 1, N = 28, p<0.001) and species (VI_self_: F = 7.93, df = 3, N = 28, p<0.001), but also an interaction between both factors on the valence rating (VI_self_: F = 106.73, df = 2.45, N = 28, p<0.001, Figure 3a). This indicates that context had different effects on the VI_self_ depending on the species. As a break down analysis we used one-sample t-tests to analyze whether the VI_self_ was significantly different from zero indicating induced positive emotional response (positive VI_self_) or negative emotional response (negative VI_self_) for each playback category (Figure 3a). Results showed that participants rated to be affected by the respective emotional valence listening to affiliative and agonistic human infant voices (t(27)≥|9.02|, N = 28, p<0.001, p_corr_<0.001). In contrast there were mixed results for the animal taxa. Participants rated to be affected by the negative emotional valence of the agonistic dog voices only (t(27) = −5.07, N = 28, p<0.001, p_corr_<0.001). The affiliative and agonistic chimpanzee voices, as well as the affiliative dog voices neither induced a positive nor a negative emotional valence in participants (t(27)≤|1.71|, N = 28, p≥0.098). Interestingly, the tree shrew voices induced the contrary emotional valence. Thus, for agonistic tree shrew voices the VI_self_ was positive (t(27) = 3.34, N = 28, p = 0.002 p_corr_ = 0.016), whereas for affiliative tree shrew voices the VI_self_ was negative (t(27)  = −3.16, N = 28, p = 0.004, p_corr_ = 0.032).

### Others-perspective

A two-factorial repeated measurement ANOVA revealed significant main effects of context (VI_other_: F = 205.08, df = 1, N = 28, p<0.001) and species (VI_other_: F = 24.37, df = 2.07, N = 28, p<0.001), but also an interaction between both factors on the valence rating (VI_other_: F = 91.37, df = 2.19, N = 28, p<0.001, Figure 3b). This indicates that context had different effects on the subjects' ratings depending on the species.

Results showed that participants classified the emotional valence correctly for agonistic and affiliative human infant voices (t(27)≥|20.16|, N = 28, p<0.001, p_corr_<0.001, Figure 3b). Again, results for the animal taxa were mixed. Participants classified the emotional valence of dog and chimpanzee voices correctly when produced in an agonistic context (t(27)≥|5.72|, N = 28, p<0.001, p_corr_<0.001), but ratings of affiliative voices did not differ from zero (t(27)≤|1.65|, N = 28, p≥0.110). Tree shrew voices were classified wrongly. Participants classified the agonistic tree shrew voices as having been produced in a positive emotional context (t(27) = 2.97, N = 28, p = 0.006, p_corr_ = 0.048) and affiliative tree shrew voices as having been produced in a negative emotional context (t(27) = −2.26, N = 28, p = 0.032, not significant after Bonferroni correction p_corr_ = 0.256).

### Self- versus others-perspective

For the valence ratings a three-factorial repeated measurement ANOVA revealed significant main effects of perspective (F = 4.76, df = 1, N = 28, p<0.038), context (F = 149.05, df = 1, N = 28, p<0.001) and species (F = 17.55, df = 3, N = 28, p<0.001), but also an interaction between these three factors (F = 9.80, df = 2.38, N = 28, p<0.001). This indicated that perspective had different effects on participants' VI depending on species and context. Therefore, we investigated the effect of perspective for each playback category separately using dependent t-tests. Results showed significant differences between VI_self_ and VI_other_ for agonistic human infant, dog and chimpanzee and affiliative chimpanzee voices (t(27)≥|3.00|, N = 28, p≤0.006, p_corr_≤0.048), for affiliative dog voices significant differences disappeared after applying Bonferroni correction (t(27) = 2.28, N = 28, p = 0.030, p_corr_ = 0.240). No significant differences were obtained for affiliative human infant and affiliative and agonistic tree shrew voices (t(27)≤|1.81|, N = 28, p≥0.082, p_corr_≥0.656). For agonistic human infant, chimpanzee and dog voices the VI_other_ was significantly more negative than the VI_self_, whereas affiliative chimpanzee voices produced the reverse pattern. Although there were significant differences for some playback categories, we found a positive correlation between VI_self_ and VI_other_ across the eight playback categories (r = 0.906, N = 8, p = 0.002).

### Influence of familiarity and self-perspective on cross-taxa emotional recognition

We found a significant positive correlation across the playback categories between the ECI and the species recognition index (objective familiarity: r  = 0.716, N = 8, p = 0.046) and between the ECI and the interaction time, i.e. time spent with the respective species (r = 0.820, N = 8, p = 0.013), emphasizing the link between familiarity and cross-taxa emotional recognition.). In contrast, there was no correlation with the assumed familiarity (r = 0.623, N = 8, p = 0.099).

We found no correlation between the VI_self_ and the ECI (r = −0.094, N = 8, p = 0.825), indicating that both are not linked.

## Discussion

Our findings provide evidence that adult male human listeners are able to recognize the emotional valence of human and some but not all animal voices. Of the investigated animal species, only the emotional valence of agonistic dog and chimpanzee voices were classified correctly. Notably tree shrew voices were classified to the contrary emotional valence. This pattern of results can be best explained by familiarity with the respective call type and context. In almost all cases where the species of the playback category was correctly recognized participants were also able to classify the emotional valence of the recording correctly (exception: affiliative dog voices). Based on the present findings reflections of induced affective states (self-perspective) or degree of phylogenetic relatedness towards humans seems to be less important for cross-taxa emotional recognition.

Human listeners classified the emotional valence of human infant voices with the highest accuracy, this being in agreement with findings in the literature (e.g., [7], [8], [9], [10], [53]). Belin et al. [7] argued that the more accurate classification of human voices could potentially rely on the selection of playback stimuli. However, in our study the same person selected both playback stimuli of human and animal voices based on the same criteria. For animal voices we found not only differences in the recognition accuracy of the emotional valence between species but also between agonistic and affiliative contexts within a species. In the following we will discuss our results for the others-perspective for each animal species and context separately.

Agonistic dog voices were correctly recognized by participants which is in agreement with findings in the literature for both call types, barks [31], [39], [40], [41]) and growls [42]. A lack of correct recognition of the affiliative dog voices was also found by Pongrácz et al. [31] for barks recorded in the ball context (i.e. the owner held a ball in front of the dog), whereas in the play context (i.e. owner played usual games with the dog such as tug-of-war, chasing or wrestling) Mudi-dog-owners (i.e. owners of dogs belonging to the Mudi breed, the breed from which playback stimuli were recorded) and non-owners were able to discriminate the play barks on the used emotional scales (e.g., playfulness, happiness). The fact that dogs use the same call type, the bark, in both, agonistic and affiliative contexts, may have made it more difficult for human listeners to discriminate the emotional valence. However, a study by Yin & McCowan already showed differences in the acoustic structure of barks recorded in various contexts [54].

For primate voices, there are only few studies investigating how humans perceive the emotional content of their voices and these provide inconsistent results. Linnankoski and colleagues [33] showed that adults and children are able to recognize the context of macaque voices correctly. In contrast, Belin and colleagues [7] did not find correct emotional classifications of rhesus monkey voices. Martin and Clark [55] played screams of chimpanzees to newborn human infants. Whereas they started to cry when listening to other newborn infant cries they did not cry when listening to chimpanzee infant cries. For the affiliative chimpanzee voices Davila Ross and colleagues [24] could reconstruct the phylogenetic tree of humans and apes based on increasing similarities in acoustic features of ape laughter, which underlines the close relatedness of the human and primate voices used in this study. However, the fact that participants did not recognize the emotional valence of affiliative chimpanzee vocalizations shows that acoustic similarities are not sufficient for explicit cross-taxa emotional recognition.

For tree shrew voices, participants were not able to classify the emotional valence correctly. Instead, they classified the contrary emotional valence. A potential explanation for these results may be the different associations participants reported (see Table S1). Participants labeled agonistic tree shrew voices mainly as birds. Sometimes they also described the stimuli as a sea-gull crying at the beach. Thus, positive associations (e.g., bird singing, sea gull on the beach) may have induced a positive emotion in the participants (self-perspective) which may then have led to the positive valence ratings for the others-perspectives. In contrast affiliative tree shrew voices were associated with the noise of a horse-drawn carriage or of the street, sounds of machines or a squeaking wheel. These sounds may have been perceived as unpleasant explaining the negative valence scores for the self-perspective. Thus it could be argued that if participants did not recognize an animated/social interaction in the sound, they may have rated the pleasantness of the stimulus rather than the emotional valence.

Our findings that agonistic chimpanzee voices did not induce negative or positive emotions (self-perspective) but were classified correctly to the negative emotional context (others-perspective) and the fact that there was no correlation between the VI_self_ and the ECI contradicts our hypothesis that a simple reflection of the self-perspective alone is sufficient for voice-induced emotional recognition. Furthermore, we found quantitative differences between the self- and the others-perspective indicating that participants reported more negative valence scores in the others- than in the self-perspective for human and animal voices. Nevertheless, valence indices of both perspectives showed a strong correlation to each other. This might be possibly because human listeners perceive these voices as less behaviorally relevant for themselves than for the sender and might be able to differentiate between how they feel when listening to the calls compared to how the other was feeling when calling. All in all, these results show that at least in human men, voice-induced recognition of emotions cannot be exhaustively explained by a simple reflection of the recipient's inner state. Further studies have to clarify to which extent cognitive processes influence the self- and others-perspective and to which extent an initial emotional response triggered by vocalizations (self-perspective) may be overridden by other cognitive mechanisms to differentiate the own emotional feeling (emotion or emotional intensity) from that of the sender.

Our findings do support the hypothesis that familiarity has a high impact on voice-induced cross-taxa recognition (others-perspective) at least in explicit rating tasks as the one used in the present study. Previous behavioral studies indicated that familiarity/experience has only little influence on cross-taxa emotional recognition. However, these studies presented only voices of one domestic animal species, which are all, to some extent, familiar with humans (e.g., cat: [37]; dog: [31]; pigs: [32]). Using this within-species design they showed that even participants who were scarcely familiar with pets (i.e. non-pet owners), blind participants and 6-month-old babies were able to recognize the emotional content of animal vocalizations correctly (e.g., cat: [37]; dog: [30], [31], [39], [43]; pig: [32]). In contrast to these studies the present study tested animal voices of different species which varied in the degree of familiarity to human listeners. By testing an absolutely unknown species, the tree shrew, we showed that familiarity does play a role in emotional recognition across species. This is pointed out in particular by the fact that participants classified the contrary emotional valence which can best be explained by cognitive associations based on similarity to or pleasantness of more well-known sounds. In previous studies familiarity was measured either as what we refer to here as assumed familiarity [37] or as frequency of interaction with the respective species (e.g., owner, non-owner, professional: [31], [32], [37], [43]). The fact that we found discrepancies between the assumed familiarity and the objective familiarity measurement shows that the former approach is problematic. In our study, humans assumed to be familiar with the respective acoustic stimuli, resulting in high assumed familiarity ratings, whereas they were in fact not able to identify the species correctly. Furthermore, our results for the chimpanzee showed that even within the same species familiarity can differ between contexts. Whereas participants recognized a primate voice as such when listening to agonistic chimpanzee voices they were not able to recognize a primate species when listening to affiliative chimpanzee voices. Thus, also the measurement of frequency of interaction may not reflect the familiarity with the call type ( = call type familiarity). We suggest that this is due to the fact that chimpanzee screams are very loud and frequently produced calls that may be encountered in zoo settings or in the media. In contrast, chimpanzee laughter is very soft, cannot be heard in a zoo settings and is only rarely displayed in the media. Furthermore, after the experiment was finished we informed the participants about the nature of the vocalizations, and almost all of them were surprised to learn that chimpanzees can produce such laughter sounds at all. This suggests that familiarity with the respective species alone is not sufficient for voice-induced cross-taxa emotional recognition. Human listeners also had to be familiar with the specific sound. Comparing the results of objective familiarity index with the classification of the recording context (others-perspective) revealed that when participants recognized the species they also recognized the emotional valence of the recording context (except for affiliative dog voices). In the case of affiliative chimpanzee voices this became especially prominent for one participant who recognized all affiliative chimpanzee voices (100%). This participant also classified all affiliative chimpanzee voices correctly (VI_other_  = 1.54). He turned out to be a biology student who had taken part in a biology course investigating chimpanzee behavior one week before the experiment. This example shows that the current results were widely influenced by experience-based cognitive mechanisms. Altogether, the present results showed a high impact of call type familiarity on voice-induced cross-taxa emotional recognition. Thus, the correct classification of the emotional valence of animal voices seems to depend on both the recognition of the species and the call type/context. Based on the discrepancy between assumed and objective familiarity it can be assumed that participants based their emotional ratings on this in part wrongly assumed familiarity, which is yet another indication of experience-based recognition mechanisms.

Our data may provide little evidence of evolutionary retained mechanisms in explicit cross-taxa emotional recognition from voice (others-perspective), at least for adult men. If phylogeny was a decisive factor, we would have expected a high recognition accuracy of emotional valence for human and chimpanzee but not for dog and tree shrew voices. This was not the case. However, an aspect of the present data that can be linked to evolutionary mechanism is that cross-taxa emotional recognition was most successful for contexts of negative emotional valence, i.e. contexts bearing high survival costs. Agonistic animal voices were better recognized than affiliative animal voices. This was also the case for pig [32] and dog vocalizations [31]. It could be argued that negative voices are more meaningful in cross-taxa communication since they convey information about possible dangerous or aggressive situations (e.g., alarm or threat calls), whereas affiliative voices are mainly used for intra-species communication (e.g. mating or contact calls). This would suggest that the acoustic structure of negative voices is evolutionarily more conserved than that of positive voices which could explain the lack of valence recognition for affiliative dog and chimpanzee voices in contrast to agonistic dog and chimpanzee voices. For dog vocalizations we have to keep in mind that domestication may have changed barking behavior such as acoustic parameters (e.g., fundamental frequency, tonality, call rate) or barking in novel contexts [16], [41], [56]. To minimize breed-specific vocal behavior, we used vocalizations from different breeds including small- and large-bodied dog breeds. However, we cannot exclude that evolutionary mechanisms are masked by domestication.

We acknowledge that different mechanisms may account for each species (e.g., domestication for dogs, pleasantness of the sound for tree shrews). However, in interpreting the results we did not just focus on one species but tried to find the most parsimonious interpretation taking all the results into account. Therefore we argue that call type familiarity has the most important impact explaining our results. It could be argued that when listening to familiar species/call type participants recognized the correct context and therefore were able to rate the valence correctly. When listening to unfamiliar stimuli participants made erroneous context associations resulting in a wrong valence rating or may have rated the others-perspective according to the self-perspective or to the pleasantness of the stimulus. The present findings may be limited by the fact that we can only assume the emotional state of an animal. In the present study we chose two superordinate context categories: affiliative context (assumed to be associated with positive emotions) and agonistic context (assumed to be associated with negative emotions). Based on video and audio analyses we related each vocalization to a special behavior of the sender (Table 1) and assigned these contexts to one of the two superordinate context categories, affiliative or agonistic context. We cannot rule out that a lack of correct recognition can also be explained by the fact that the animal is not in the assumed emotional state and therefore the receiver has no chance to recognize the context. To solve this problem, comparative acoustical designs are necessary to test the perception of conspecific and heterospecific species in humans and animals using the same acoustic stimuli.

## Conclusions

In conclusion, adult human male listeners showed highest emotional recognition accuracy for conspecific voices, while the recognition accuracy towards animal voices depended mainly on call type familiarity, i.e. the recognition of the species and the respective call type/context. These findings suggest that at least under explicit task conditions cross-taxa voice-induced emotional recognition in adult men is more affected by cognitive experience-based mechanisms than by phylogeny. Further studies have to investigate whether these results can be extended to women and infants/children, and to what extent such cognitive processes can mask the perception of possible universal cues in mammalian vocalizations, and whether an implicit approach to the processing of other species' emotional voices is more suitable for revealing evolutionarily retained mechanisms. Currently, an EEG and an fMRI study are under way to investigate the temporal determinants and neuronal networks underlying cross-taxa voice-induced emotional perception.

## Supporting Information

Table S1 Response categories related to the question “What kind of a sound was it?”.
(XLS)
